# Genome Sequence and Methylome of Soil Bacterium *Gemmatirosa kalamazoonensis* KBS708^T^, a Member of the Rarely Cultivated *Gemmatimonadetes* Phylum

**DOI:** 10.1128/genomeA.00226-14

**Published:** 2014-04-03

**Authors:** Jennifer M. DeBruyn, Mark Radosevich, K. Eric Wommack, Shawn W. Polson, Loren J. Hauser, Mariam N. Fawaz, Jonas Korlach, Yu-Chih Tsai

**Affiliations:** a Department of Biosystems Engineering & Soil Science, University of Tennessee, Knoxville, Tennessee, USA; b Department of Plant & Soil Science, University of Delaware, Newark, Delaware, USA; c Center for Bioinformatics & Computational Biology, University of Delaware, Newark, Delaware, USA; d Oak Ridge National Laboratory, Oak Ridge, Tennessee, USA; e Pacific Biosciences, Menlo Park, California, USA

## Abstract

Bacteria belonging to the phylum *Gemmatimonadetes* are found in a wide variety of environments and are particularly abundant in soils. Here, we present the complete genome sequence and methylation pattern of the newly described *Gemmatirosa kalamazoonensis* type strain.

## GENOME ANNOUNCEMENT

Bacteria belonging to the phylum *Gemmatimonadetes* are frequently found in soils (1). To date, only two *Gemmatimonadetes* strains have been characterized: *Gemmatimonas aurantiaca* T-27 from wastewater (2) and *Gemmatirosa kalamazoonensis* KBS708 isolated from soil (3). Here, we report the complete genome sequence of *G. kalamazoonensis*.

*G. kalamazoonensis* KBS708^T^ (ATCC BAA-2150, NCCB 100411) was grown for 10 days on VL55 minimal medium with 0.025% peptone, as previously described (3). Genomic DNA was extracted using an UltraClean microbial DNA isolation kit (Mo Bio) and randomly sheared to ~10-kb target size using G-tubes (Covaris, Inc.). Poly(dA) tails were added to the 3′ ends using terminal deoxynucleotidyl transferase (TdT). The poly(dA)-tailed library was then annealed with poly(dT) sequencing primer and sequenced using DNA/polymerase binding kit 2.0 with a MagBead loading kit and 120-min sequencing time on the PacBio RS instrument (Pacific Biosciences, Inc.).

Single-molecule real-time (SMRT) sequencing data collected using the TdT library at Pacific Biosciences was combined with standard SMRT sequencing data collected at the University of Delaware for *de novo* genome assembly using the Hierarchical Genome Assembly Process (HGAP) (4). Initial output from HGAP yielded one 5,318-kb chromosome (118× coverage) and two large satellite elements (1,059 kb and 1,041 kb with 102× and 98× coverage, respectively). A collapsed 53-kb tandem repeat region in the 1,059-kb element was identified and resolved into a 1,106-kb element. A 21-kb high-copy element with sequence overlapping the chromosome was also resolved in the process (376× coverage). This manual curation process resulted in a 5,312-kb circular chromosome and three circular satellite elements (1,106 kb, 1,041 kb, and 21 kb), with 72.6% average G+C content. Final assembly was polished using the Quiver consensus algorithm included in the SMRT analysis software package. Base modifications were identified using the base modification analysis protocol (Pacific Biosciences).

The Prodigal genome annotation pipeline at Oak Ridge National Laboratory (5, 6) was used to predict genes and provide annotation based on homology searches. A total of 6,373 candidate protein-coding genes were predicted. The 5.3-Mb chromosome contained 48 tRNA genes. Two sets of *rrn* genes were identified, with one set in an operon and the second split, as its 16S rRNA gene was located 1.28 Mb away and on the opposite strand from the 23S-5S genes. A total of 4,515 protein coding genes were predicted, 3,434 of which were assigned a function based on homology. The two large plasmids (1.1 Mb and 1.0 Mb) contain 1,026 and 798 predicted genes, respectively. The small high-copy element (21 kb) contains 34 predicted genes; the 5 genes that were assigned functions suggest it may be a phage.

Methylation analysis (7) revealed the presence of three active N^6^-methyladenine methyltransferases with the recognition sequences 5′-RG**A**TCY-3′, 5′-ATGC**A**C-3′, and 5′-CC**A**GN_7_TCA-3′, each with >99% of the genomic positions conforming to the sequence motif detected as methylated (boldface denotes a methylated base; underlining denotes a methylated base on the opposite DNA strand).

### Accession numbers.

The genome sequences and methylation data are deposited at NCBI under GenBank accession no. CP007127 to CP007130 and GEO accession no. GSE55390.
